# Urging health collaboration to combat antimicrobial resistance between China and B&R countries

**DOI:** 10.1186/s40249-022-01033-4

**Published:** 2022-10-17

**Authors:** Zile Cheng, Nan Zhou, Xiaoxi Zhang, Chao Lv, Xiaokui Guo, Yongzhang Zhu, Min Li

**Affiliations:** 1 Department of Animal Health and Food Safety, School of Global Health, Chinese Center for Tropical Diseases Research, Shanghai Jiao Tong University School of Medicine, Shanghai, 200025 China; 2 One Health Center, Shanghai Jiao Tong University, The University of Edinburgh, Shanghai, 200025 China

## Background

Antimicrobial resistance (AMR) was regarded as one of the top ten global public health problems by the World Health Organization (WHO). While the complexity of AMR poses threats to humans, animals, and the environment, to combat AMR necessitates a multi-sector approach. In March 2022, world leaders and experts called on all countries to reduce antimicrobial pollution to protect the environment, and WHO published the strategic framework for collaboration on AMR in May, emphasizing significant global threat of AMR and calling for multisectoral coordination based on One Health approach [1, 2].

In 2013, China proposed the construction of the Silk Road Economic Belt and the 21st Century Maritime Silk Road (the Belt and Road, B&R). As of July 2022, 149 countries and 32 international organizations participated in the Belt and Road Initiative (BRI) [3]. BRI attached considerable priority to health, and the Boao Forum for Asia in 2021 focused on new forms of health cooperation for “Healthy Silk Road” initiative [4]. However, AMR-related aspects have not been involved in B&R health cooperation projects. As the proponent of B&R, China need to enhance AMR-related health collaboration. This report is to recommend detailed strategies or plans for further enhancing the AMR governance capabilities in B&R, based on a comprehensive assessment of the current AMR status according to the Global One Health Index for AMR (GOHI-AMR) [5].

### Current AMR status of B&R countries

With the GOHI-AMR platform, which is the first One Health-based systemic tool to conduct a global assessment of AMR, we would be able to assess the AMR status and provide specific recommendations for the B&R’s continued health implementation strategies or plans.

There were 149 countries in B&R, of which 113 countries were included in the GOHI-AMR database including China [5]. They were geographically grouped into 6 primary regions based on World Bank classification standards: 16 countries in East Asia and the Pacific (EAP, average scores: 43.44), 33 countries in Europe and Central Asia (ECA, 42.30), 12 countries in Latin America and Caribbean (LAC, 34.21), 14 countries in Middle East and North Africa (MENA, 37.31), 5 countries in South Asia (SA, 32.23), and 33 countries in Sub-Saharan Africa (SSA, 29.99). It could be seen Sub-Saharan Africa’s scores were lower and there were significant discrepancies across B&R countries (Additional file 1: Fig. S1A). As shown in Additional file 1: Fig. S1B, comparing the GOHI-AMR sub-scores between the six groups (detailed data in Additional file 2: Fig. S2), the most significant metric difference was NTC indicator, showing a huge gap in the establishment of the national reference laboratory system on AMR. And most of the disparity indicators were related to SSA, which reflected in SSA high antimicrobial consumption in animal, incomplete action plan and national laws. Surprisingly, there was high prevalence of AMR for Aminoglycosides in ECA.

### Recommendations

Given the large AMR discrepancies between B&R countries indicated by GOHI-AMR data, the future health cooperation plan in B&R should prioritize the most critical AMR concerns. Due to the close relationship between AMR and the human living environment, we focused on the One Health approach and propose the following recommendations for the current primary situation:

i. Increasing One Health-based funds and talent: This may necessitate a greater emphasis on the cultivation and exchange plans of talent in One Health-related fields by subsidizing education funds and the recruitment of more pharmaceutical companies to increase capital investment.
ii. Establishing a monitoring network of antimicrobial consumption: Linking countries to build a surveillance platform of antimicrobial consumption and use, especially on antimicrobial abuse, comprehensively monitoring and regularly reporting the overall consumption of humans, animals and pesticides, urging high-consuming countries to rationally optimize the management of antibiotic usage.
iii. Establishing collaborative laboratories to monitor AMR: Utilizing the joint laboratory platform, standardize AMR surveillance plan, facilitate reciprocal exchanges on AMR management, organize regular professional technical training, invite countries with relatively complete AMR management to share their experiences, and improve the national action plan for AMR surveillance.
iv. Establishing a cross-border AMR surveillance online database in B&R: It is essential to establish online AMR surveillance data based on the joint laboratory. Thus, nations can stay aware of the emerging AMR trends and bridge the gap between them.
v. Strengthening ecological governance continuously: Combined with the Belt and Road Ecological and Environmental Cooperation Plan, adhering to the concept of One Health, promote implementation of the 2030 Agenda for Sustainable Development, which must prioritize monitoring AMR in the environment and in animals. Continually boost investment in AMR-related ecological prevention research, including vaccine, probiotics, lactic acid bacteria, and enzybiotics (Additional file 2).

## Conclusions

AMR has remained a global public health concern, and its effects vary considerably in B&R countries. Countries should adopt the One Health strategy, pay great attention to AMR issues, provide financial and human resource assistance, utilize joint laboratories and a cross-border AMR monitoring database to promote health cooperation, and prioritize ecological governance in accordance with other B&R cooperation plans. This report's suggestions serve as a guide for future B&R public health cooperation and policy development.

## Supplementary Information

**Additional file 1.** Fig.S1.

**Additional file 2.** Fig.S2.

## Data Availability

All the data came from the reference [5].

## Abbreviations

AMR: Antimicrobial resistance
BRI: The Belt and Road Initiative
B&R: The Belt and Road
GOHI-AMR: Global One Health index for Antimicrobial Resistance
EAP: East Asia and the Pacific
ECA: Europe and Central Asia
LAC: Latin America and Caribbean
NTC: National AMR capacity
SA: South Asia
SSA: Sub-Saharan Africa
