# Supplementary figures and images for: Urging health collaboration to combat antimicrobial resistance between China and B&R countries
Source: Infect Dis Poverty. 2022 Oct 17;11:108. doi: 10.1186/s40249-022-01033-4 (PMC9575246; doi:10.1186/s40249-022-01033-4)

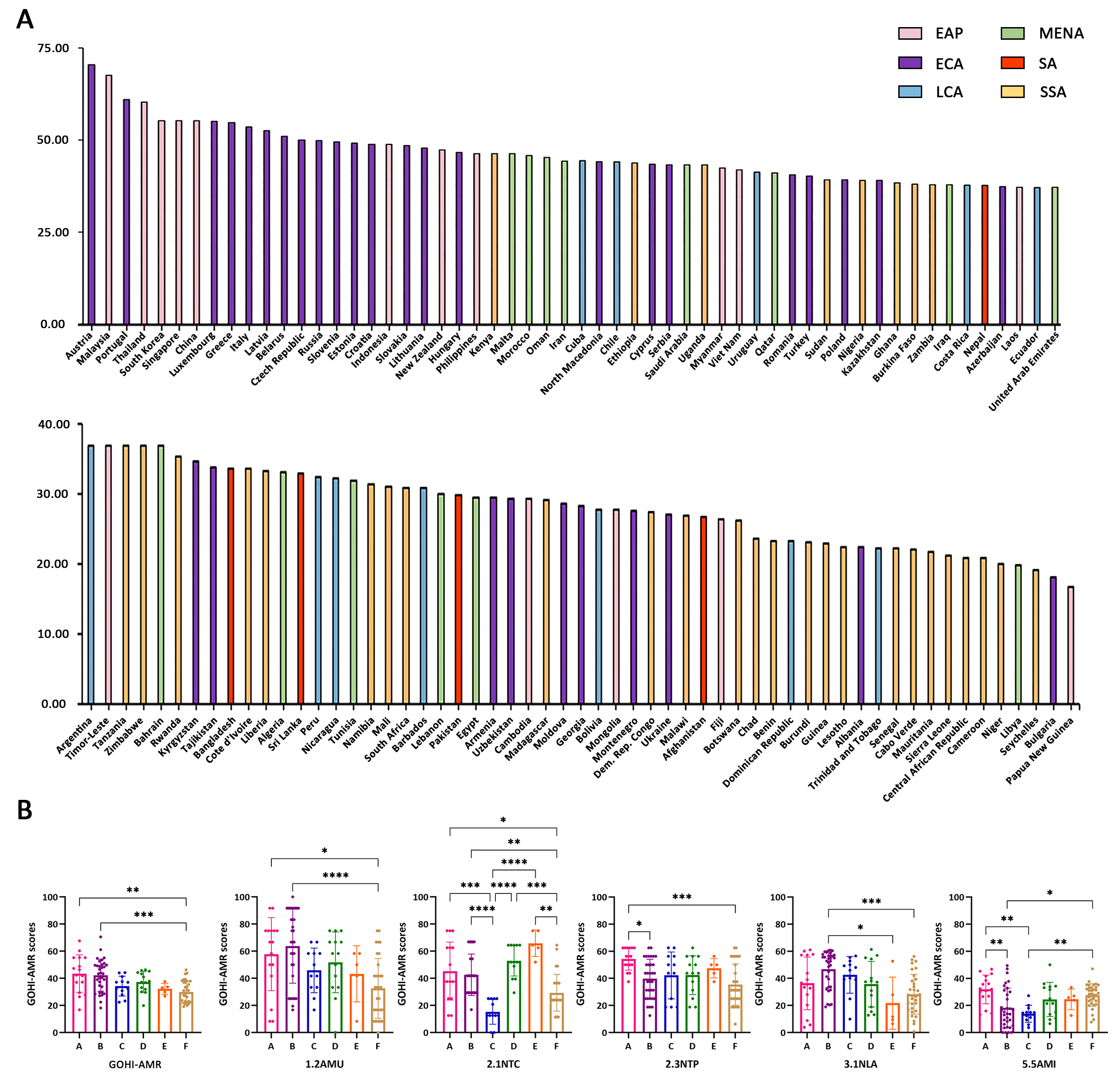

Supplement: Supplementary file 1 — Additional file 1. Fig.S1. [file 40249_2022_1033_MOESM1_ESM.jpg]

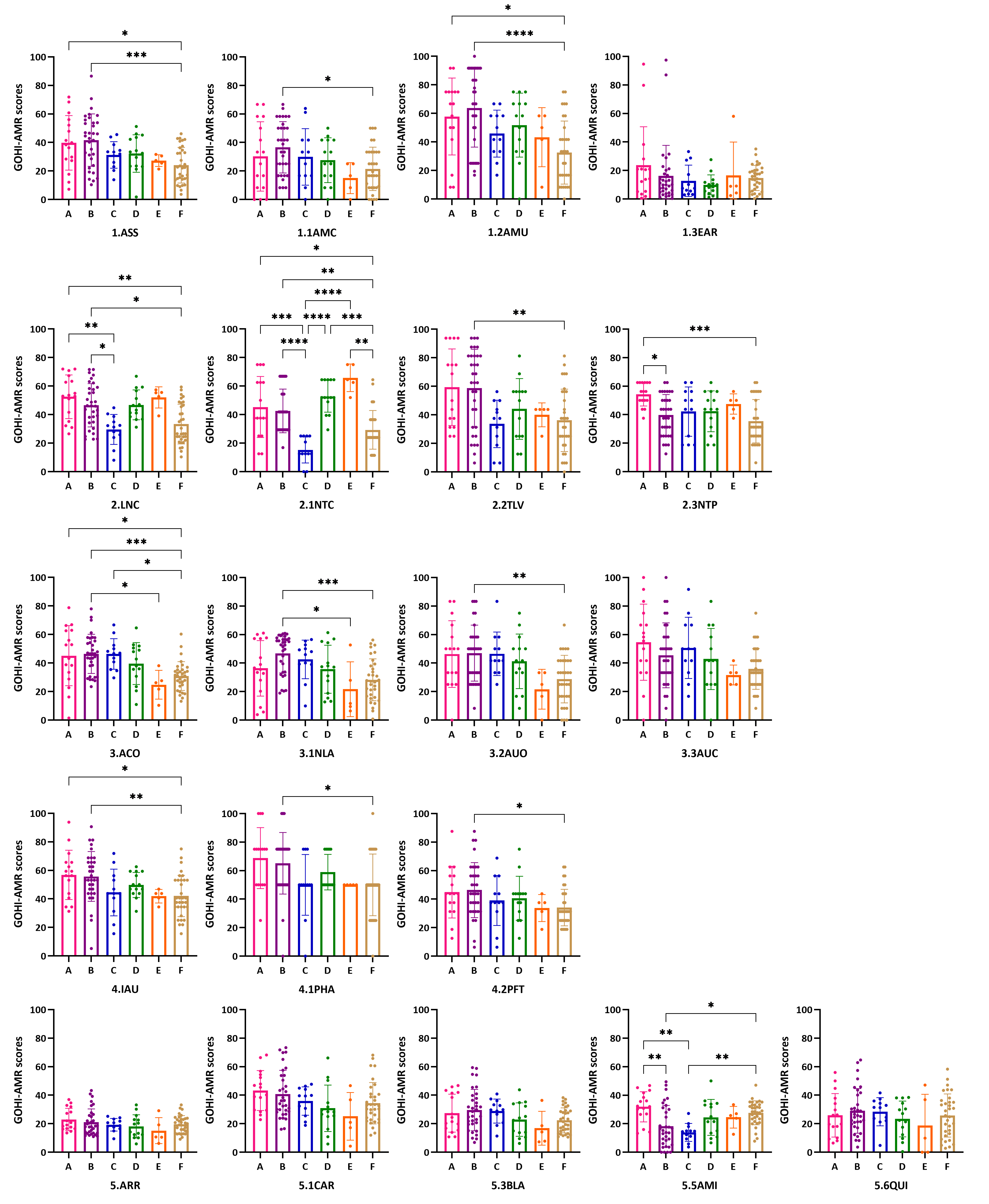

Supplement: Supplementary file 2 — Additional file 2. Fig.S2. [file 40249_2022_1033_MOESM2_ESM.jpg]
